# Root-Associated Fungi Shared Between Arbuscular Mycorrhizal and Ectomycorrhizal Conifers in a Temperate Forest

**DOI:** 10.3389/fmicb.2018.00433

**Published:** 2018-03-12

**Authors:** Hirokazu Toju, Hirotoshi Sato

**Affiliations:** ^1^Center for Ecological Research, Kyoto University, Otsu, Japan; ^2^Precursory Research for Embryonic Science and Technology, Japan Science and Technology Agency, Kawaguchi, Japan; ^3^Graduate School of Human and Environmental Studies, Kyoto University, Kyoto, Japan

**Keywords:** arbuscular mycorrhizal fungi, Chaetothyriales, dark septate endophytes, ectomycorrhizal fungi, ecological communities, Helotiales, host specificity, Illumina

## Abstract

Arbuscular mycorrhizal and ectomycorrhizal symbioses are among the most important drivers of terrestrial ecosystem dynamics. Historically, the two types of symbioses have been investigated separately because arbuscular mycorrhizal and ectomycorrhizal plant species are considered to host discrete sets of fungal symbionts (i.e., arbuscular mycorrhizal and ectomycorrhizal fungi, respectively). Nonetheless, recent studies based on high-throughput DNA sequencing technologies have suggested that diverse non-mycorrhizal fungi (e.g., endophytic fungi) with broad host ranges play roles in relationships between arbuscular mycorrhizal and ectomycorrhizal plant species in forest ecosystems. By analyzing an Illumina sequencing dataset of root-associated fungi in a temperate forest in Japan, we statistically examined whether co-occurring arbuscular mycorrhizal (*Chamaecyparis obtusa*) and ectomycorrhizal (*Pinus densiflora*) plant species could share non-mycorrhizal fungal communities. Among the 919 fungal operational taxonomic units (OTUs) detected, OTUs in various taxonomic lineages were statistically designated as “generalists,” which associated commonly with both coniferous species. The list of the generalists included fungi in the genera *Meliniomyces, Oidiodendron, Cladophialophora, Rhizodermea, Penicillium*, and *Mortierella*. Meanwhile, our statistical analysis also detected fungi preferentially associated with *Chamaecyparis* (e.g., *Pezicula*) or *Pinus* (e.g., *Neolecta*). Overall, this study provides a basis for future studies on how arbuscular mycorrhizal and ectomycorrhizal plant species interactively drive community- or ecosystem-scale processes. The physiological functions of the fungi highlighted in our host-preference analysis deserve intensive investigations for understanding their roles in plant endosphere and rhizosphere.

## Introduction

In terrestrial ecosystems, most plant species form intimate interactions with mycorrhizal fungi, which play essential roles in the growth and survival of their hosts (van der Heijden et al., 2008; Bever et al., 2010; Peay et al., 2016). Those fungi, for example, supply soil nitrogen and phosphorous to associated plants, thereby enhancing hosts' physiological states (Smith and Read, 2008). They are also known to reduce deleterious effects of pathogens on host plants (Marx, 1972; Azcón-Aguilar and Barea, 1997; Borowicz, 2001). Moreover, mycorrhizal fungi can contribute to physiological homeostasis of plants by increasing hosts' resistance to abiotic stress (Grover et al., 2011). Therefore, understanding and managing below-ground integrations between plants and their mycorrhizal fungal symbionts are major challenges not only in basic ecology but also in forestry and agronomy.

Among the several categories of mycorrhizal fungi, arbuscular mycorrhizal, and ectomycorrhizal fungi are major groups of below-ground fungal communities in temperate forests (Smith and Read, 2008; Peay et al., 2016). Arbuscular mycorrhizal fungi (the phylum Glomeromycota) first appeared early in the history of land plants (Remy et al., 1994) and hence they associate with plant species in diverse plant taxa (Schüβler et al., 2001). They are obligate mutualistic symbionts and hence rely entirely on carbon supply from host plants (Smith and Read, 2008). While they are abundant in root systems of herbaceous plants (Hiiesalu et al., 2014), they are hosted also by diverse tree species (Liu et al., 2015). Ectomycorrhizal fungi, which consist mainly of the phyla Ascomycota and Basidiomycota, appeared in the era of seed plant diversification (Hibbett and Matheny, 2009). In contrast to arbuscular mycorrhizal fungi, some of them may obtain carbon not only from plants but also from soil by decomposing dead organic matter (Talbot et al., 2008) (but see Lindahl and Tunlid, 2015). Ectomycorrhizal fungi play important roles in forest community dynamics because they promote the dominance of the specific plant families (e.g., Pinaceae, Fagaceae, Betulaceae, and Dipterocarpaceae; Tedersoo et al., 2010; Tedersoo and Smith, 2013) through “positive plant–soil feedbacks” (Booth, 2004; McGuire, 2007; Bennett et al., 2017). Due to the difference in their major host taxa, arbuscular mycorrhizal and ectomycorrhizal fungi have been considered to form distinct sets of symbioses with their arbuscular mycorrhizal plant and ectomycorrhizal plant hosts (Smith and Read, 2008), potentially driving discrete community ecological dynamics. As a consequence, arbuscular mycorrhizal and ectomycorrhizal symbioses have been investigated separately in most mycological studies.

Nonetheless, recent studies integrating high-throughput DNA sequencing and host–symbiont network analyses have shown that diverse non-mycorrhizal fungi with broad host ranges are associated with roots of both arbuscular mycorrhizal and ectomycorrhizal plants within terrestrial ecosystems (Toju et al., 2014a, 2015). Furthermore, mycorrhizal, endophytic, and other types of root-associated fungi have been reported to co-occur within/around a tiny segment of plant roots (Read and Haselwandter, 1981; Mandyam and Jumpponen, 2005; Nakamura et al., 2017), potentially interacting with each other positively or negatively (Toju et al., 2016b) (cf. Kennedy et al., 2009; Werner and Kiers, 2015). Interestingly, an increasing number of studies have shown that non-mycorrhizal fungi (e.g., endophytic fungi) can supply host plants with phosphorous, potentially playing physiological roles similar to those of mycorrhizal fungi (Jumpponen, 2001; Narisawa et al., 2002; Newsham, 2011; Hiruma et al., 2016; Almario et al., 2017). Thus, host plant ranges of those non-mycorrhizal fungi are of particular interest because they will provide a basis for uncovering potential sharing of soil nutrients between arbuscular mycorrhizal and ectomycorrhizal plants and its consequences on the community- or ecosystem-level dynamics (Kadowaki et al., in review). However, while an increasing number of studies have evaluated host preferences (or generality) of diverse functional groups of root-associated fungi including possible endophytes (Huang et al., 2008; Kernaghan and Patriquin, 2011; Botnen et al., 2014; Sato et al., 2015), most studies have investigated either arbuscular mycorrhizal or ectomycorrhizal plant species but not both. Consequently, we still have limited knowledge of how co-occurring arbuscular mycorrhizal and ectomycorrhizal plant species can interact with each other indirectly through below-ground webs of symbioses involving not only mycorrhizal but also diverse non-mycorrhizal fungi.

In this study, we statistically examined host preferences of not only mycorrhizal but also root-endophytic fungi in a mixed forest of arbuscular mycorrhizal and ectomycorrhizal coniferous trees in Japan. We sampled roots of *Chamaecyparis obtusa* (arbuscular mycorrhizal) and *Pinus densiflora* (ectomycorrhizal) and then revealed community compositions of the fungi associated with the two plant species based on Illumina sequencing. The dataset allowed us to classify those fungi in terms of their host preferences, highlighting endophytic fungi preferentially found from either *Chamaecyparis* or *Pinus*, and those commonly associated with both plant species. Overall, this study provides a basis for future studies examining how diverse functional groups of below-ground fungi mediate interactions between arbuscular mycorrhizal and ectomycorrhizal plant species in terrestrial ecosystems.

## Materials and methods

### Sampling

Fieldwork was conducted in a secondary temperate forest in Sasayama, Hyogo Prefecture, Japan (35.094 °N, 135.238 °E) on June 6, 2016. Sampling in the forest was permitted by the committee of the local residents. Within the forest consisted mainly of *Pinus densiflora* (Pineaceae), *Quercus serrata* (Fagaceae), and *Ilex pedunculosa* (Aquifoliaceae), there were patches of planted *Chamaecyparis obtuse* (Cupressaceae). Along a mountain trail in the forest, we collected 2 cm segments of terminal roots at 3 cm below the soil surface at 1 m horizontal intervals, screening coniferous tree roots based on root morphology: angiosperm roots were excluded in the sampling. In total, 247 root samples were collected and delivered to the laboratory within the sampling day. The samples were stored at −80°C until DNA extraction.

### DNA extraction, PCR, and sequencing

Each root sample was washed in 80% ethanol by sonication for 5 min. DNA extraction was then performed with a cetyltrimethylammonium bromide (CTAB) method (Sato and Murakami, 2008). We amplified the internal transcribed spacer 1 (ITS1) region of root-associated fungi using the forward primer ITS1F-KYO1 (Toju et al., 2012) fused with 3–6-mer Ns for improved Illumina sequencing quality (Lundberg et al., 2013) and the forward Illumina sequencing primer (5′- TCG TCG GCA GCG TCA GAT GTG TAT AAG AGA CAG- [3–6-mer Ns]–[ITS1-KYO2]−3′) and the reverse primer ITS2-KYO2 (Toju et al., 2012) fused with 3–6-mer Ns and the reverse sequencing primer (5′- GTC TCG TGG GCT CGG AGA TGT GTA TAA GAG ACA G [3–6-mer Ns]—[ITS2_KYO2]−3′). In the PCR, the buffer and DNA polymerase kit of KOD FX Neo (Toyobo) was used with a temperature profile of 94°C for 2 min, followed by 35 cycles at 98°C for 10 s, 50°C for 30 s, 68°C for 50 s, and a final extension at 68 °C for 5 min. The ramp rate through the thermal cycles was set to 1°C/s in order to prevent generation of chimeric sequences (Stevens et al., 2013). To add Illumina sequencing adaptors to respective samples, supplemental PCR was performed using the forward fusion primers consisting of the P5 Illumina adaptor, 8-mer indexes for sample identification (Hamady et al., 2008), and a partial sequence of the sequencing primer (5′- AAT GAT ACG GCG ACC ACC GAG ATC TAC AC—[8-mer index]—TCG TCG GCA GCG TC−3′) and the reverse fusion primers consisting of the P7 adaptor, 8-mer indexes, and a partial sequence of the sequencing primer (5′- CAA GCA GAA GAC GGC ATA CGA GAT—[8-mer index]—GTC TCG TGG GCT CGG−3′). KOD FX Neo was used with a temperature profile of 94°C for 2 min, followed by 8 cycles at 98°C for 10 s, 55°C for 30 s, 68°C for 50 s (ramp rate = 1°C/s), and a final extension at 68°C for 5 min. The PCR amplicons of the 247 root samples and a negative control sample were pooled with equal volume after a purification/equalization process with AMPureXP Kit (Beckman Coulter). The ratio of AMPure reagent to amplicons was set to 0.6 (v/v) in order to remove primer dimers (i.e., sequences shorter than 200 bp).

To discriminate *Chamaecyparis* and *Pinus* root samples, we performed, another set of PCR targeting plant chloroplast *rbcL* region using the rbcL_F3 and rbcL_R4 primers (Toju et al., 2013a) with the same DNA polymerase system, temperature profiles, and purification processes used in the fungal ITS analysis. The sequencing libraries of fungal ITS and plant *rbcL* regions were processed in an Illumina MiSeq sequencer (run center: KYOTO-HE) with the 2 × 250 cycle sequencing kit (20% PhiX spike-in).

### Bioinformatics

The raw sequencing data were converted into FASTQ files using the program bcl2fastq 1.8.4 distributed by Illumina. The output FASTQ files were demultiplexed with the program Claident v0.2.2016.07.05 (Tanabe and Toju, 2013; Tanabe, 2016). Sequencing reads whose 8-mer index positions included nucleotides with low (<30) quality scores were removed in this process. Given that the quality of reverse Illumina sequences is generally much lower than that of forward sequences, only forward sequences were used after removing low-quality 3'-ends using Claident: the sequencing data are available on the DNA Data Bank of Japan (DDBJ) (DDBJ Sequence Read Archive accession; DRA006340). Noisy reads (Tanabe, 2016) were subsequently discarded and then 2,177,205 ITS and 92,013 *rbcL* filtered reads were obtained.

For the analysis of the ITS region, filtered reads were clustered with the program VSEARCH (Rognes et al., 2016) as implemented in Claident. Taking into account the high intraspecific ITS-sequence variation of Glomeromycota (Thiéry et al., 2016), the cut-off sequence similarity in the clustering of the fungal ITS region was set to 95%. The molecular identification of the output 1183 OTUs (Supplementary Data 1) was conducted based on the combination of the query-centric auto-*k*-nearest neighbor (QCauto) method (Tanabe and Toju, 2013) and the lowest common ancestor (LCA) algorithm (Huson et al., 2007) as implemented in Claident. Note that taxonomic identification results based on the combination of the QCauto search and the LCA taxonomic assignment are comparable to, or sometimes more accurate than, those with the alternative approach combining the UCLUST algorithm (Edgar, 2010) with the UNITE database (Kõljalg et al., 2013) (see Toju et al., 2016a,b for detailed comparison between the QCauto-LCA and UCLUST–UNITE approaches). The functional group of each fungal OTU was inferred using the program FUNGuild 1.1 (Nguyen et al., 2016) (Supplementary Data 2). As the FUNGuild program often output multiple guilds for a single OTU (e.g., “Ectomycorrhizal-Orchid Mycorrhizal-Root Associated Biotroph”), the output guild information was grouped into the following categories in light of Tedersoo et al. (2010) and Smith and Read (2008): i.e., arbuscular mycorrhizal, ectomycorrhizal, ericoid mycorrhizal, saprotrophic/endophytic, plant pathogenic, animal pathogenic, and unclassified fungi (see Supplementary Data 2 for details). Given that fungi in the endosphere often shift their lifestyle when host plant tissue dies (Porras-Alfaro and Bayman, 2011), we grouped potentially endophytic and saprotrophic fungal OTUs into a single category (saprotrophic/endophytic).

The Illumina sequences of the plant *rbcL* region were processed with a cut-off sequence similarity of 97%. Based on the taxonomic assignment results with Claident, *Chamaecyparis* and *Pinus* samples were discriminated: four samples turned out to be angiosperm roots were discarded. We then obtained a sample × fungal OTU matrix, in which a cell entry depicted the number of sequencing reads of an OTU in a sample. The cell entries whose read counts represented less than 0.1% of the total read count of each sample were removed to minimize the effects of PCR/sequencing errors (Peay et al., 2015). The filtered matrix was then rarefied to 2,000 reads per sample using the “rrarefy” function of the vegan 2.4-1 package (Oksanen et al., 2012) of R 3.3.2 (R-Core-Team, 2015). The samples with less than 2,000 reads and the 264 fungal OTUs with no read counts in the rarefied matrix were eliminated. We then obtained a matrix consisting of 208 root samples (157 *Chamaecyparis* and 51 *Pinus* samples) and 919 fungal OTUs (Supplementary Data 3).

### Fungal diversity

For all the statistical analyses below, the vegan package of R was used. We first examined relationship between the number of sequencing reads and that of detected fungal OTUs with the “rarecurve” function. Likewise, relationship between the number of root samples and that of fungal OTUs was visualized with the “specaccum” function. Root-associated fungal community compositions were then compared between *Chamaecyparis* and *Pinus*, focusing on the functional groups and order-level taxonomy of observed fungi. Difference in fungal community compositions between the two plant species was further examined by the permutational analysis of variance (Anderson, 2001) with the “adonis” function (PERMANOVA; 10,000 permutations). We also performed the permutational analysis for the multivariate homogeneity of dispersions (PERMDISP) (Anderson, 2006) with the “betadisper” function. Differentiation of fungal community structure between *Chamaecyparis* and *Pinus* was also examined by non-metric multidimensional scaling (NMDS) with the “metaMDS” function. Potential spatial autocorrelation in the fungal community data was evaluated based on a Mantel's correlogram analysis with the “mantel.correlog” function. The “Raup-Crick” metric of β-diversity (Chase et al., 2011) (“raupcrick” function) was used in the PERMANOVA, PERMDISP, NMDS, and Mantel's correlogram analyses.

### Host preference

To explore root-associated fungi showing preference for *Chamaecyparis* or *Pinus*, we performed an analysis based on the multinomial species classification method (CLAM test; Chazdon et al., 2011) with the “clamtest” function of the R vegan package. The CLAM test has been used for exploring “generalists” and “specialists” based on comparisons between contrasting habitats or host species (Toju et al., 2013b, 2014b). Importantly, the multinomial model implemented in the test minimizes biases due to differing sampling intensities between the two habitats or host species compared (Chazdon et al., 2011). Based on a CLAM test, fungal OTUs were classified into four categories: fungal OTUs displaying statistically significant preferences for *Chamaecyparis*, those with significant preference for *Pinus*, those commonly found from both plant species, and those too rare to be evaluated statistically.

## Results

### Fungal diversity

**Figure 1:**
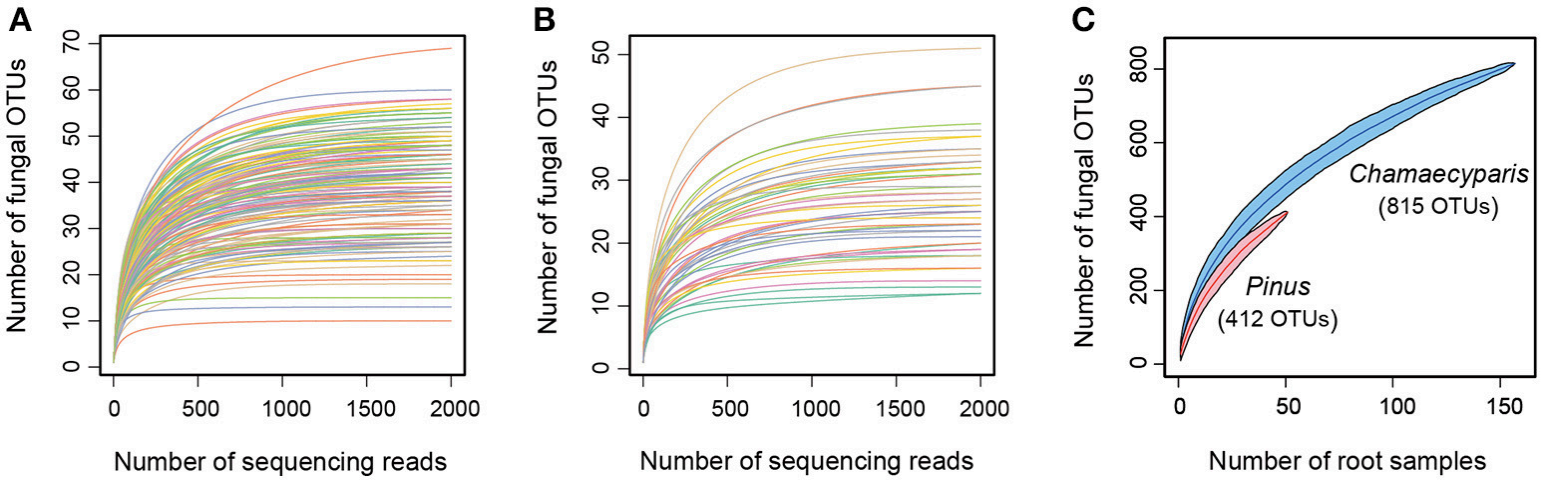
Fungal OTU richness. **(A)** Relationship between the number of sequencing reads and that of fungal OTUs (*Chamaecyparis* root samples). **(B)** Relationship between the number of sequencing reads and that of fungal OTUs (*Pinus* root samples). **(C)** Relationship between the number of root samples and that of fungal OTUs.

**Figure 2:**
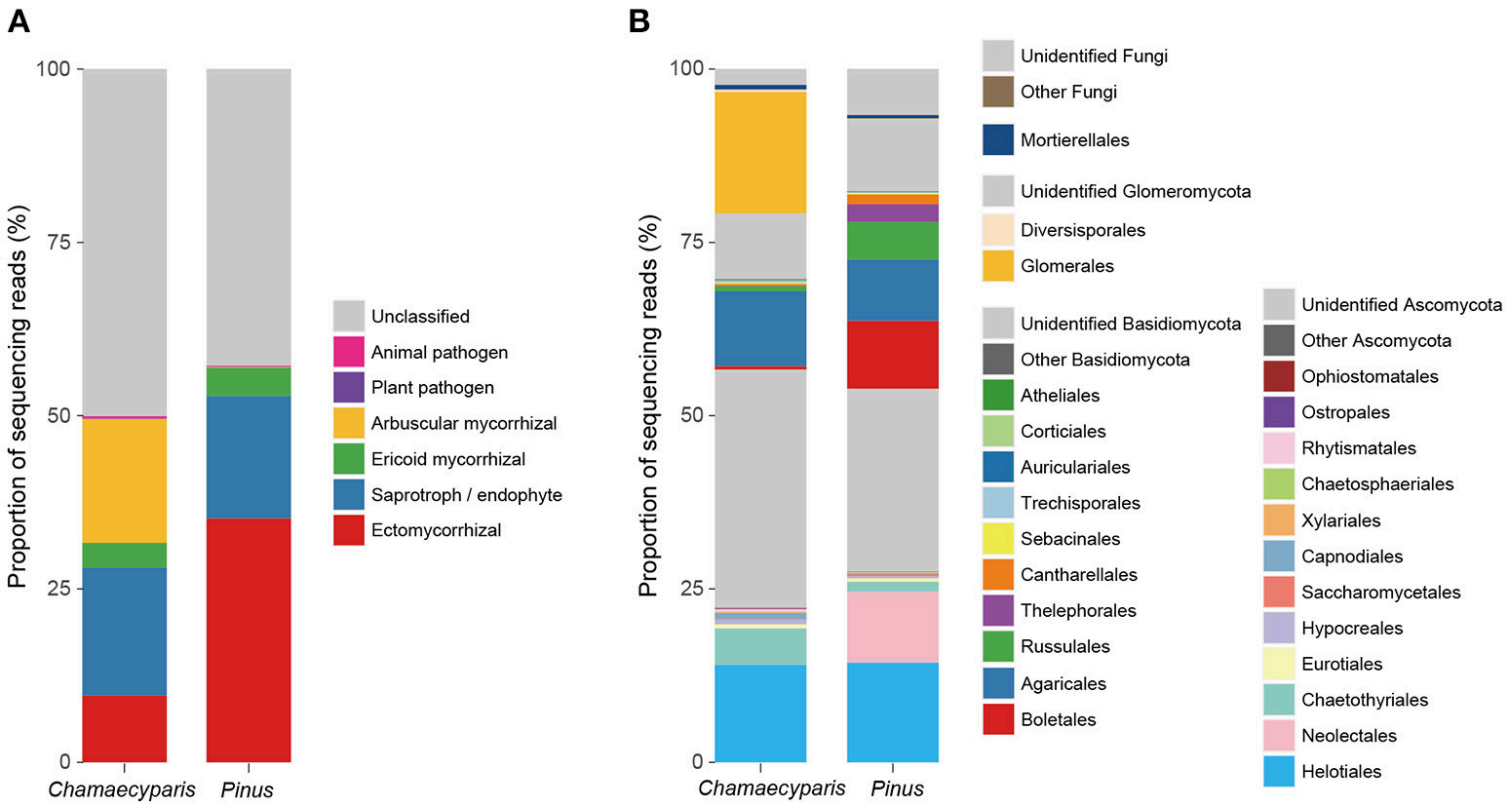
Compositions of the fungal community. **(A)** Functional groups (category). Average proportions of sequencing reads were calculated for *Chamaecyparis* and *Pinus* root samples. **(B)** Order-level taxonomy.

**Figure 3:**
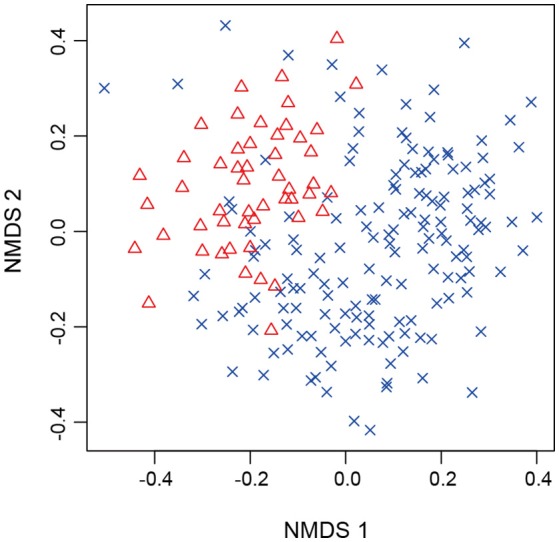
NMDS of root samples. *Chamaecyparis* (cross) and *Pinus* (triangle) samples were plotted on a NMDS surface (stress = 0.288).

**Figure 4:**
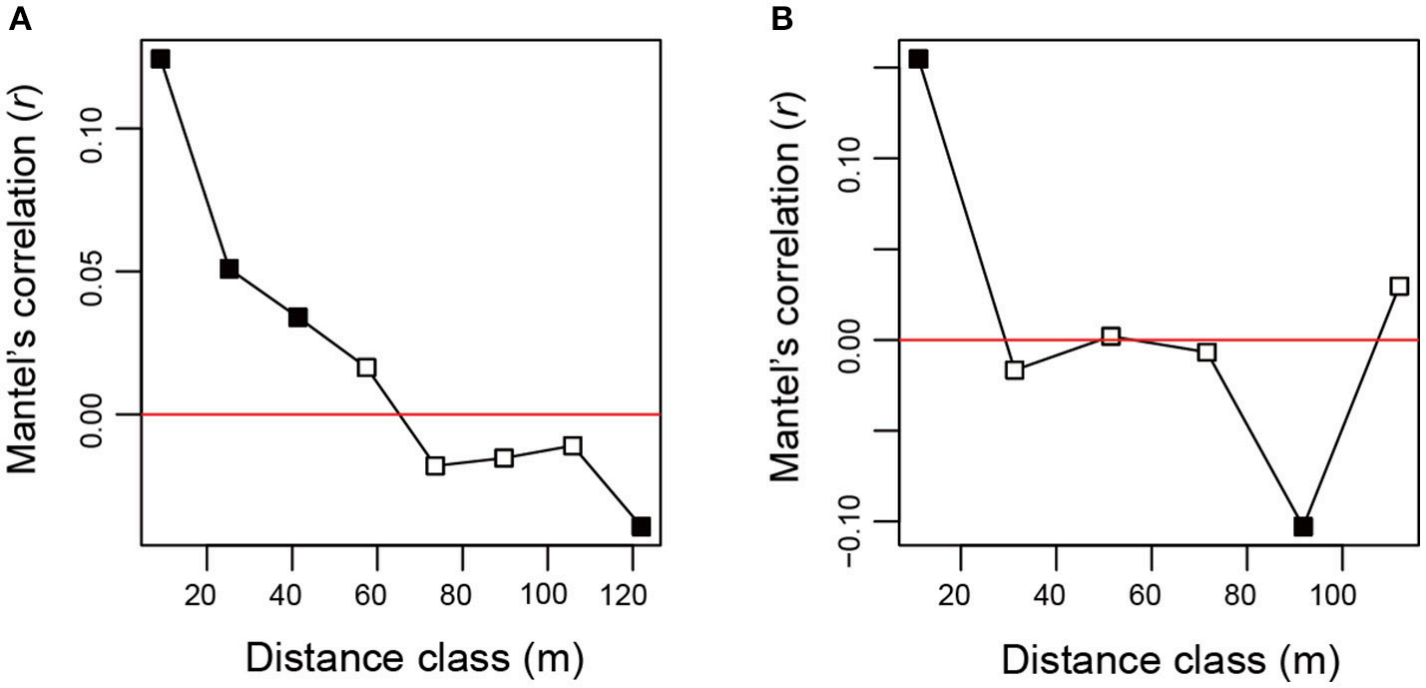
Spatial autocorrelation of fungal community structure. **(A)** Mantel's correlogram analysis of *Chamaecyparis* samples. A positive value indicated by filled squares represents statistically significant spatial autocorrelation at the spatial distance class (α = 0.05). **(B)** Mantel's correlogram analysis of *Pinus* samples.

After a series of bioinformatics and rarefaction process, 815 and 412 fungal OTUs were found from *Chamaecyparis* and *Pinus*, respectively (Figure 1). The fungal community compositions differed between the two species. For example, arbuscular mycorrhizal fungi occurred almost exclusively on *Chamaecyparis*, while ectomycorrhizal and saprotrophic/endophytic fungi occurred on both plant species (Figure 2A). Regarding order-level taxonomy, Helotiales, Chaetothyriales, Agaricales, and Glomerales occurred frequently on *Chamaecyparis* (Figure 2B). In contrast, the root-associated fungal community of *Pinus* was characterized by Neolectales, Boletales, Russulales, and Thelephorales, although it resembled the *Chamaecyparis* fungal community in terms of Helotiales relative abundance (Figure 2B). The differentiation of fungal community structure between the two plant species was statistically significant (PERMANOVA; df = 1, *F*_model_ = 58.4, *P* < 0.0001) (Figure 3), although the structural difference was attributed, at least partly, to the heterogeneity of among-sample variation (PERMDISP; df = 1, *F* = 9.4, *P* = 0.003). The structure of root-associated fungal communities displayed spatial autocorrelation within 50 m and 20 m in *Chamaecyparis* and *Pinus*, respectively (Figure 4).

### Host preference

**Figure 5:**
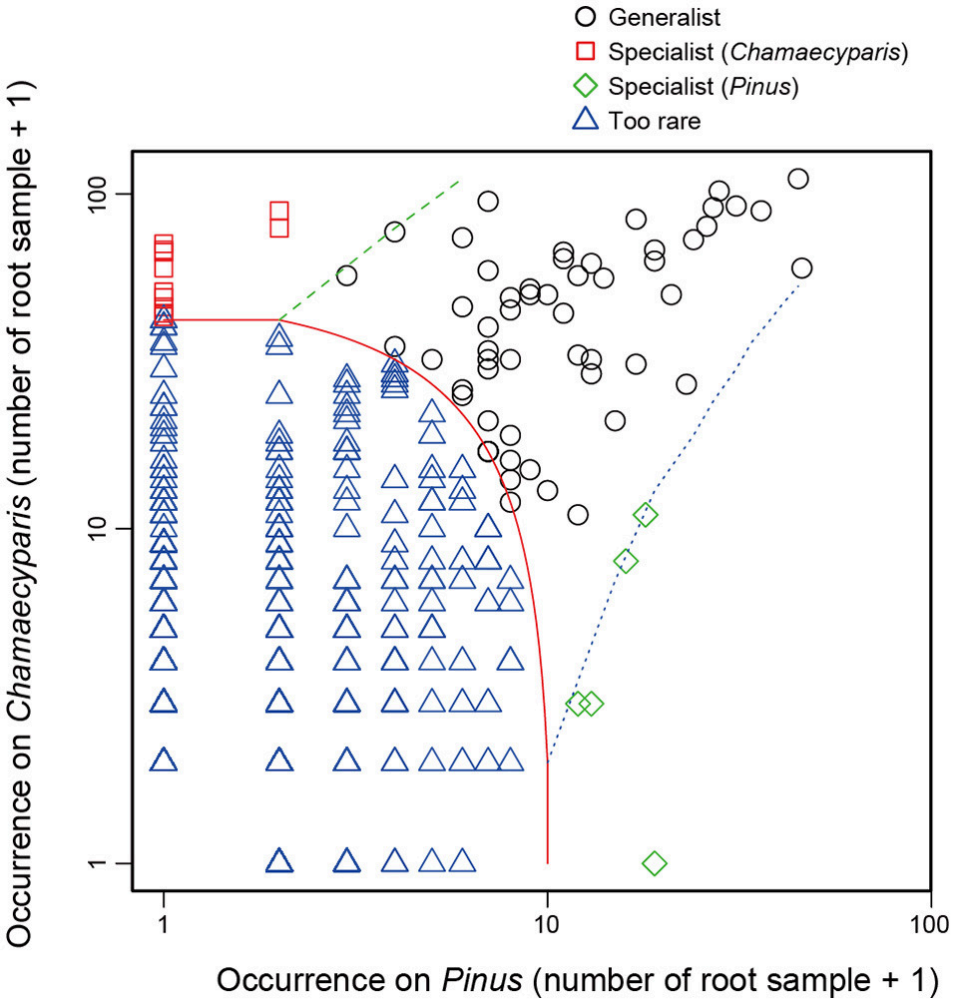
Screening of specialists and generalists. Fungal OTUs commonly detected from both *Chamaecyparis* and *Pinus* root samples (circle), those preferentially found from *Chamaecyparis* (square) or *Pinus* (diamond) samples, and rare fungal OTUs (triangle) were classified by a CLAM test.

In the CLAM test (Figure 5), fungal OTUs in various taxonomic lineages were classified as “generalists,” meaning fungi commonly found from both plant species (Table 1). Among them, a *Meliniomyces* fungus in the order Helotiales appeared in 70.1% (110/157) and 86.3% (44/51) of *Chamaecyparis* and *Pinus* samples, respectively (Table 1). The *Meliniomyces* OTU was allied to *M. variabilis* (NCBI accession: HM190129), which were reported as saprotrophic, endophytic, and ericoid mycorrhizal but not ectomycorrhizal (Vrålstad et al., 2002a,b; Hambleton and Sigler, 2005): note that all *Meliniomyces* fungal OTUs were automatically designated as “ectomycorrhizal” by the FUNGuild program used in this study. The ascomycetous genera *Oidiodendron, Cladophialophora, Rhizodermea*, and *Penicillium* were also commonly found from the two coniferous species (Table 1). The statistical test also highlighted fungal OTUs showing host preferences for *Chamaecyparis* or *Pinus* (Table 2). A fungus in the genus *Pezicula* (Helotiales) and 10 glomeromycete OTUs were classified as OTUs associated preferentially with *Chamaecyparis*. In contrast, *Neolecta* sp. (Neolectales), Dermataceae sp. (Helotiales), and three other fungi were preferentially associated with *Pinus*.

**Table 1:** Fungal OTUs commonly found from both *Chamaecyparis* and *Pinus* roots.

**OTU**	***N* (*Cham*.)**	***N* (*Pinus*)**	**Phylum**	**Class**	**Order**	**Family**	**Genus**	**Category**
F_001	110	44	Ascomycota	Leotiomycetes	Helotiales	Helotiaceae	*Meliniomyces*	EcM
F_017	101	27	Ascomycota	–	–	–	–	–
F_010	91	30	Ascomycota	–	–	–	–	–
F_004	90	26	Ascomycota	–	–	–	–	–
F_005	88	35	Ascomycota	Leotiomycetes	–	Myxotrichaceae	*Oidiodendron*	ErM
F_006	83	16	Ascomycota	Eurotiomycetes	Chaetothyriales	Herpotrichiellaceae	*Cladophialophora*	SapEndo
F_054	79	25	Ascomycota	Leotiomycetes	–	–	–	–
F_022	72	23	Ascomycota	–	–	–	–	–
F_018	67	18	Ascomycota	–	–	–	–	–
F_015	66	10	Basidiomycota	Agaricomycetes	–	–	–	–
F_014	63	10	Ascomycota	–	–	–	–	–
F_021	62	18	–	–	–	–	–	–
F_051	61	12	Ascomycota	Leotiomycetes	Helotiales	Dermateaceae	*Rhizodermea*	SapEndo
F_016	59	45	–	–	–	–	–	–
F_106	56	11	–	–	Mortierellales	Mortierellaceae	*Mortierella*	SapEndo
F_080	55	13	Ascomycota	Eurotiomycetes	Eurotiales	Aspergillaceae	*Penicillium*	SapEndo
F_071	49	20	Ascomycota	Eurotiomycetes	Eurotiales	Aspergillaceae	–	SapEndo
F_125	43	10	–	–	Mortierellales	Mortierellaceae	*Mortierella*	SapEndo
F_131	32	11	–	–	Mortierellales	Mortierellaceae	*Mortierella*	SapEndo
F_048	31	12	Ascomycota	–	–	–	–	–
F_029	30	16	Ascomycota	–	–	–	–	–
F_113	28	12	Basidiomycota	Tremellomycetes	–	–	–	–
F_045	26	22	Ascomycota	Leotiomycetes	–	Myxotrichaceae	*Oidiodendron*	ErM
F_046	20	14	Ascomycota	–	–	–	–	–
F_019	10	11	Basidiomycota	Agaricomycetes	Boletales	Rhizopogonaceae	*Rhizopogon*	EcM

*In a CLAM test, Chamaecyparis and Pinus root samples (157 and 51 samples, respectively) were analyzed to explore “generalist” fungal OTUs, which were commonly associated with both Chamaecyparis and Pinus. For simplicity, “generalist” fungal OTUs that occurred in less than 10 Chamaecyparis or Pinus samples are omitted from the list above. The number of Chamaecyparis/Pinus samples from which each fungal OTU was observed is shown for each OTU. EcM, ectomycorrhizal; ErM, ericoid mycorrhizal; SapEndo, saprotrophic or endophytic*.

**Table 2:** Fungal OTUs showing statistically significant host preferences.

**OTU**	***N* (*Cham*.)**	***N* (*Pinus*)**	**Phylum**	**Class**	**Order**	**Family**	**Genus**	**Category**
***CHAMAECYPARIS***								
F_020	88	1	Glomeromycota	Glomeromycetes	Glomerales	Glomeraceae	*Glomus*	AM
F_039	78	1	Glomeromycota	Glomeromycetes	Glomerales	Glomeraceae	*Glomus*	AM
F_036	70	0	Glomeromycota	Glomeromycetes	Glomerales	Glomeraceae	*Rhizophagus*	AM
F_052	67	0	Glomeromycota	Glomeromycetes	Glomerales	Glomeraceae	–	AM
F_038	66	0	Glomeromycota	Glomeromycetes	Glomerales	Glomeraceae	–	AM
F_034	59	0	Glomeromycota	Glomeromycetes	Glomerales	Glomeraceae	–	AM
F_088	50	0	Glomeromycota	Glomeromycetes	Glomerales	Glomeraceae	–	AM
F_092	48	0	Glomeromycota	Glomeromycetes	Glomerales	Glomeraceae	–	AM
F_072	45	0	Ascomycota	Leotiomycetes	Helotiales	Dermateaceae	*Pezicula*	SapEndo
F_147	43	0	Glomeromycota	Glomeromycetes	Glomerales	Glomeraceae	*Glomus*	AM
F_175	42	0	Glomeromycota	Glomeromycetes	Glomerales	Glomeraceae	–	AM
***PINUS***								
F_009	0	18	Ascomycota	Neolectomycetes	Neolectales	Neolectaceae	*Neolecta*	SapEndo
F_139	10	17	Ascomycota	Leotiomycetes	–	–	–	–
F_013	7	15	Basidiomycota	Agaricomycetes	–	–	–	–
F_150	2	12	Ascomycota	Leotiomycetes	Helotiales	Dermateaceae	–	–
F_123	2	11	Ascomycota	Dothideomycetes	–	–	–	–

*Fungal OTUs showing preferences for Chamaecyparis or Pinus were indicated by a CLAM test. The number of Chamaecyparis/Pinus samples from which each fungal OTU was observed is shown for each OTU. AM, arbuscular mycorrhizal; SapEndo, saprotrophic or endophytic*.

Although the difference in sample size between *Chamaecyparis* and *Pinus* (157 and 51 samples, respectively) might have caused biases in the statistical analyses conducted in this study, results qualitatively similar with the abovementioned analyses were obtained in a series of supplementary analyses with equalized sample size (i.e., 51 randomly chosen *Chamaecyparis* samples vs. 51 *Pinus* samples) (Supplementary Figures 1–3; Supplementary Tables 1–2).

## Discussion

Our data provided a novel opportunity to compare mycorrhizal and non-mycorrhizal fungal communities between arbuscular mycorrhizal (*Chamaecyparis*) and ectomycorrhizal (*Pinus*) plants in a temperate forest. One of the recent conceptual advances in mycology is that plant species in the wild interact not only with mycorrhizal fungi but also with diverse taxonomic/functional groups of endosphere and rhizosphere fungi (Mandyam and Jumpponen, 2005; Newsham, 2011). Those recent findings challenge the classic view that plant species differing in mycorrhizal type form discrete sets of below-ground plant–fungus interactions. Hereafter, we discuss fungi potentially mediating arbuscular mycorrhizal and ectomycorrhizal symbioses as well as those that preferentially interact with either mycorrhizal type of plant hosts.

Many of the fungi found commonly from both plant species belonged to major orders in Ascomycota, namely, Helotiales, Chaetothyriales, and Eurotiales (Figure 2B; Table 1). Among them, *Meliniomyces* (Helotiales) (Hambleton and Sigler, 2005) showed surprisingly high infection rates, appearing in 70 and 86% of *Chamaecyparis* and *Pinus* root samples, respectively. Although some species in *Meliniomyces–Rhizoscyphus* complex have been confirmed to be ectomycorrhizal in pure culture synthetic trials, the most abundant OTU detected in this study was allied to *M. variabilis*, which has been inferred as saprotrophic, endophytic, or ericoid mycorrhizal (Vrålstad et al., 2002a,b; Hambleton and Sigler, 2005; Tedersoo et al., 2009). Interestingly, *M. variabilis* obtained from a Norway spruce (*Picea abies*) microhabitats lacking ericaceous plants formed ericoid mycorrhizae with European blueberry (*Vaccinium myrtillus*) under experimental conditions, promoting the growth of the host (Vohník et al., 2013). Another Helotiales fungus frequently detected from both *Chamaecyparis* and *Pinus* roots belonged to the genus *Rhizodermea*. A fungus in the genus has been reported to enhance heavy-metal stress tolerance of host plants (Yamaji et al., 2016). Our analysis also detected *Cladophialophora* (Chaetothyriales), a lineage of so-called “dark septate endophytes” (Jumpponen and Trappe, 1998; Jumpponen, 2001). A species in the genus (*C. chaetospira*) has been known to enhance growth and pathogen resistance of host plants (Morita et al., 2003; Usuki and Narisawa, 2007). *Penicillium* (Eurotiales) fungi are also reported frequently from roots of diverse plant taxa, although they are generally considered as saprotrophic soil fungi (Watanabe, 2010) or postharvest pathogens of fruits (Agrios, 2005). However, given the repeated reports of *Penicillium* fungi from seemingly benign roots of diverse plant species (Cao et al., 2002; Toju et al., 2016b), some of them may play positive roles. Indeed, some *Penicillium* species associated with wheat are known to solubilize phosphorous in rhizosphere or endosphere (Wakelin et al., 2004). *Penicillium* species are also known to produce a series of antibiotics, which potentially inhibits growth of plant pathogens (Yang et al., 2008).

We also detected *Mortierella* and *Oidiodendron* fungi as common symbionts of *Chamaecyparis* and *Pinus* roots (Table 1). Fungi in the genus *Mortierella* are often isolated from soil and root systems in various types of habitats (Watanabe, 2010). Although they are generally regarded as saprotrophs, some of them potentially promote plant growth by suppressing root-knot nematodes or phytopathogens such as *Rhizoctonia* and *Cercospora* (Eroshin and Dedyukhina, 2002; AL-Shammari et al., 2013). Fungi in the genus *Oidiodendron* (anamorph of *Myxotrichum*) are also reported from diverse soil environments, while the genus include ericoid mycorrhizal fungi, *O. maius* and *O. griseum* (Couture et al., 1983; Douglas et al., 1989; Rice and Currah, 2005; Vohník et al., 2005). *Oidiodendron* fungi were also reported from roots of non-ericaceous plants such as *Betula, Picea*, and *Abies* trees in a boreal forest (Kernaghan and Patriquin, 2011).

While there were ectomycorrhizal fungi frequently detected from both *Chamaecyparis* and *Pinus* roots (*Rhizopogon*), no arbuscular mycorrhizal fungi were designated as “host generalists” in our study (Table 1). This pattern is of particular interest in light of previous studies reporting asymmetry in host–symbiont associations between ectomycorrhizal and arbuscular mycorrhizal symbioses (Plattner and Hall, 1995; Dickie et al., 2001). For example, colonization of ectomycorrhizal fungi might be deleterious to non-ectomycorrhizal plants as reported in a herbaceous plant species, whose roots suffered from severe necrosis after infection of the truffle fungus, *Tuber melanosporum* (Plattner and Hall, 1995) (see also Booth, 2004). Thus, in the forest studied in this study, the presence of *Pinus* and its ectomycorrhizal fungi may have negative impacts on *Chamaecyparis*, although possibilities that those ectomycorrhizal fungi play positive or neutral roles in *Chamaecyparis* root systems deserve further investigations. Arbuscular mycorrhizal fungi have been also reported to interact with non-typical host plant species. For example, an oak species (*Quercus rubra*) is known to host not only ectomycorrhizal but also arbuscular mycorrhizal fungi in the vicinity of arbuscular mycorrhizal plants (Dickie et al., 2001). The nearly complete absence of arbuscular mycorrhizal fungi in *Pinus* roots in our study (Figure 2A) highlights context dependency in such host–symbiont associations that span conventional categories of mycorrhizal symbioses.

The statistical analysis conducted in this study also allowed us to explore fungal species preferentially associated with either *Chamaecyparis* or *Pinus*. As expected, many arbuscular mycorrhizal fungi were found almost exclusively from *Chamaecyparis*. Meanwhile, a Helotiales fungus in the genus *Pezicula* (anamorph, *Cryptosporiopsis*; Verkley, 1999) (Chen et al., 2016) showed a strong preference for *Chamaecyparis*. Given that fungi in the genus produce secondary metabolites (e.g., mullein and echinocandin) that inhibit growth of plant pathogens (Noble et al., 1991; Schulz et al., 1995; Wang et al., 2014), *Chamaecyparis* hosts may be benefited by the presence of the endophytic fungi. Among the fungi preferentially associated with *Pinus*, an ascomycete fungus in the genus *Neolecta* (Neolectales) displayed the strongest host preference. Although their functions remain unknown, *Neolecta* fungi are known to associate with plant roots (Redhead, 1979; Landvik et al., 2003): an observation of co-occurrence of a *Neolecta* fungus and an ectomycorrhizal fungus in root tips (Redhead, 1979) is intriguing in postulating their functions. Although these results on potential host preferences are of particular ecological interest, it should be acknowledged that this study did not take into account possible spatial heterogeneity of edaphic factors (e.g., soil pH and C/N ratios) within the study site: there were too many sampling positions to perform detailed chemical analyses. In the dataset, we observed spatial autocorrelations in the occurrences of *Chamaecyparis*/*Pinus* root samples (Supplementary Figure 4) and fungal community structure (Figure 4). To evaluate relative contributions of host preference and spatial environmental heterogeneity, more sophisticated statistical analyses (e.g., latent variable model analyses; Warton et al., 2015) needs to be tried in future studies.

Our screening of plant-associated below-ground fungi with narrow/broad host ranges provides crucial implications for the understanding of dynamic linkage between plant and below-ground fungal communities (Klironomos, 2002; Bever et al., 2010; van der Putten et al., 2013). Previous studies on arbuscular mycorrhizal plants have shown “negative plant–soil feedbacks”, in which increases of host-specific soil microbes result in decline of the host plant populations (Bever, 2002; Kardol et al., 2007; Mangan et al., 2010). In contrast, positive feedbacks leading to monodominance have been suspected for interactions between ectomycorrhizal plants and their ectomycorrhizal fungi (Booth, 2004; McGuire, 2007; Bennett et al., 2017). While most of those previous studies focused on plant–soil feedbacks operating in interactions involving a single plant species and their mycorrhizal (and pathogenic) fungi, arbuscular mycorrhizal, and ectomycorrhizal fungi often coexist within a forest (Dickie et al., 2001; Toju et al., 2014a), potentially driving feedbacks across different mycorrhizal types (Kadowaki et al., in review). In this respect, the observed asymmetry in infection patterns of arbuscular mycorrhizal and ectomycorrhizal fungi (Figure 2A) helps us postulate possible directionality in such across-mycorrhizal-type dynamics of plant and below-ground fungal communities.

Also intriguingly, this study identified a number of endophytic fungi associated with both arbuscular mycorrhizal and ectomycorrhizal plants and those specific to either mycorrhizal type of plant species (Tables 1, 2; Figure 5). Given the prevalence of endophytic fungi and their functional effects on host plant growth (Jumpponen and Trappe, 1998; Jumpponen, 2001; Newsham, 2011), understanding of plant–soil feedbacks would be never complete without taking into account the entire association networks involving not only mycorrhizal but also non-mycorrhizal fungi. Among endophytic fungal taxa potentially playing pivotal roles in such plant–soil feedbacks, the ascomycete order Helotiales (Tedersoo et al., 2009; Almario et al., 2017; Nakamura et al., 2017) is of particular interest because they included not only OTUs specific to either arbuscular mycorrhizal or ectomycorrhizal plant species but also generalist OTUs associated with both categories of host plants. Experimental studies testing the roles of host-specific and generalist endophytic fungi are awaited to build frameworks for describing and forecasting forest community dynamics.

## Author contributions

HT designed the work. HT and HS performed fieldwork. HT conducted molecular experiment. HT wrote the manuscript with HS.

### Conflict of interest statement

The authors declare that the research was conducted in the absence of any commercial or financial relationships that could be construed as a potential conflict of interest.
