# Supplementary material for: Root-Associated Fungi Shared Between Arbuscular Mycorrhizal and Ectomycorrhizal Conifers in a Temperate Forest
Source: Front Microbiol. 2018 Mar 12;9:433. doi: 10.3389/fmicb.2018.00433 (PMC5858530; doi:10.3389/fmicb.2018.00433)
Supplement: Supplementary file 1 [file Table1.PDF]

Supplementary Table 1. Fungal OTUs commonly found from both *Chamaecyparis* and *Pinus* roots (analysis with equal sample size). An equal number of *Chamaecyparis* and *Pinus* root samples (51 samples each) were subjected to a CLAM test in order to explore "generalist" fungal OTUs, which were commonly associated with both *Chamaecyparis* and *Pinus*. For simplicity, "generalist" fungal OTUs that occurred in less than 10 *Chamaecyparis* or *Pinus* samples are omitted from the list below. The number of *Chamaecyparis*/*Pinus* samples from which each fungal OTU was observed is shown for each OTU. EcM, ectomycorrhizal; ErM, ericoid mycorrhizal; SapEndo, saprotrophic or endophytic.

OTU	*N* (*Cham.*)	*N* (*Pinus*)	Phylum	Class	Order	Family	Genus	Category
F_001	37	44	Ascomycota	Leotiomycetes	Helotiales	Helotiaceae	*Meliniomyces*	EcM
F_017	35	27	Ascomycota	-	-	-	-	-
F_010	34	30	Ascomycota	-	-	-	-	-
F_004	30	26	Ascomycota	-	-	-	-	-
F_005	28	35	Ascomycota	Leotiomycetes	-	Myxotrichaceae	*Oidiodendron*	ErM
F_006	28	16	Ascomycota	Eurotiomycetes	Chaetothyriales	Herpotrichiellaceae	*Cladophialophora*	SapEndo
F_018	27	18	Ascomycota	-	-	-	-	-
F_054	24	25	Ascomycota	Leotiomycetes	-	-	-	-
F_021	22	18	-	-	-	-	-	-
F_080	21	13	Ascomycota	Eurotiomycetes	Eurotiales	Aspergillaceae	*Penicillium*	SapEndo
F_106	21	11	-	-	Mortierellales	Mortierellaceae	*Mortierella*	SapEndo
F_022	20	23	Ascomycota	-	-	-	-	-
F_051	20	12	Ascomycota	Leotiomycetes	Helotiales	Dermateaceae	*Rhizodermea*	SapEndo
F_015	20	10	Basidiomycota	Agaricomycetes	-	-	-	-
F_016	18	45	-	-	-	-	-	-
F_125	16	10	-	-	Mortierellales	Mortierellaceae	*Mortierella*	SapEndo
F_014	14	10	Ascomycota	-	-	-	-	-
F_071	12	20	Ascomycota	Eurotiomycetes	Eurotiales	Aspergillaceae	-	SapEndo
