# Supplementary material for: Root-Associated Fungi Shared Between Arbuscular Mycorrhizal and Ectomycorrhizal Conifers in a Temperate Forest
Source: Front Microbiol. 2018 Mar 12;9:433. doi: 10.3389/fmicb.2018.00433 (PMC5858530; doi:10.3389/fmicb.2018.00433)
Supplement: Supplementary file 2 [file Table2.PDF]

Supplementary Table 2. Fungal OTUs showing statistically significant host preferences (analysis with equal sample size). Fungal OTUs showing preferences for *Chamaecyparis* or *Pinus* were indicated by a CLAM test. The number of *Chamaecyparis*/*Pinus* samples from which each fungal OTU was observed is shown for each OTU. AM, arbuscular mycorrhizal; SapEndo, saprotrophic or endophytic.

OTU	*N* (*Cham.*)	*N* (*Pinus*)	Phylum	Class	Order	Family	Genus	Category
Chamaecyparis								
F_020	28	1	Glomeromycota	Glomeromycetes	Glomerales	Glomeraceae	Glomus	AM
F_039	25	1	Glomeromycota	Glomeromycetes	Glomerales	Glomeraceae	Glomus	AM
F_036	22	0	Glomeromycota	Glomeromycetes	Glomerales	Glomeraceae	Rhizophagus	AM
F_038	21	0	Glomeromycota	Glomeromycetes	Glomerales	Glomeraceae	-	AM
F_052	21	0	Glomeromycota	Glomeromycetes	Glomerales	Glomeraceae	-	AM
F_092	21	0	Glomeromycota	Glomeromycetes	Glomerales	Glomeraceae	-	AM
F_034	20	0	Glomeromycota	Glomeromycetes	Glomerales	Glomeraceae	-	AM
Pinus								
F_009	0	18	Ascomycota	Neolectomycetes	Neolectales	Neolectaceae	Neolecta	SapEndo
F_013	2	15	Basidiomycota	Agaricomycetes	-	-	-	-
F_150	1	12	Ascomycota	Leotiomycetes	Helotiales	Dermateaceae	-	-
