# Supplementary material for: Root-Associated Fungi Shared Between Arbuscular Mycorrhizal and Ectomycorrhizal Conifers in a Temperate Forest
Source: Front Microbiol. 2018 Mar 12;9:433. doi: 10.3389/fmicb.2018.00433 (PMC5858530; doi:10.3389/fmicb.2018.00433)
Supplement: Supplementary file 6 [file Image1.PDF]

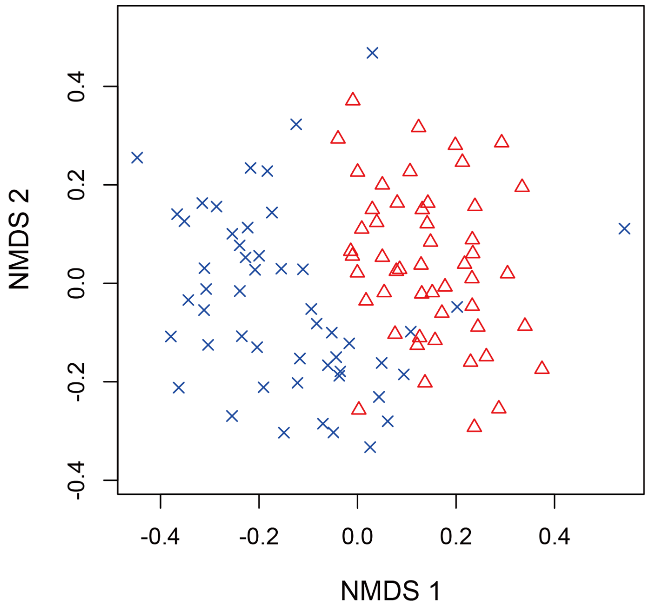

Supplementary Figure 1. NMDS of root samples (analysis with equal sample size). On a NMDS surface, 51 *Chamaecyparis* (cross) and 51 *Pinus* (triangle) samples were plotted (stress = 0.278). The differentiation of fungal community structure between the two plant species was statistically significant (PERMANOVA; df = 1, $F_{\text{model}} = 49.4$, $P < 0.0001$) (Fig. 3), although the structural difference was attributed, at least partly, to the heterogeneity of among-sample variation (PERMDISP; df =1, $F = 4.9$, $P = 0.03$).
