# Supplementary material for: Root-Associated Fungi Shared Between Arbuscular Mycorrhizal and Ectomycorrhizal Conifers in a Temperate Forest
Source: Front Microbiol. 2018 Mar 12;9:433. doi: 10.3389/fmicb.2018.00433 (PMC5858530; doi:10.3389/fmicb.2018.00433)
Supplement: Supplementary file 7 [file Image2.PDF]

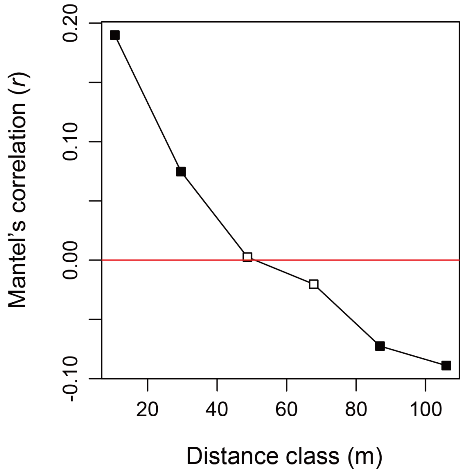

Supplementary Figure 2. Spatial autocorrelation of fungal community structure (analysis with equal sample size). Mantel's correlogram analysis for 51 subsampled *Chamaecyparis* roots. A positive value indicated by filled squares represent statistically significant spatial autocorrelation at the spatial distance class ($\alpha = 0.05$).
