# Supplementary material for: Root-Associated Fungi Shared Between Arbuscular Mycorrhizal and Ectomycorrhizal Conifers in a Temperate Forest
Source: Front Microbiol. 2018 Mar 12;9:433. doi: 10.3389/fmicb.2018.00433 (PMC5858530; doi:10.3389/fmicb.2018.00433)
Supplement: Supplementary file 8 [file Image3.PDF]

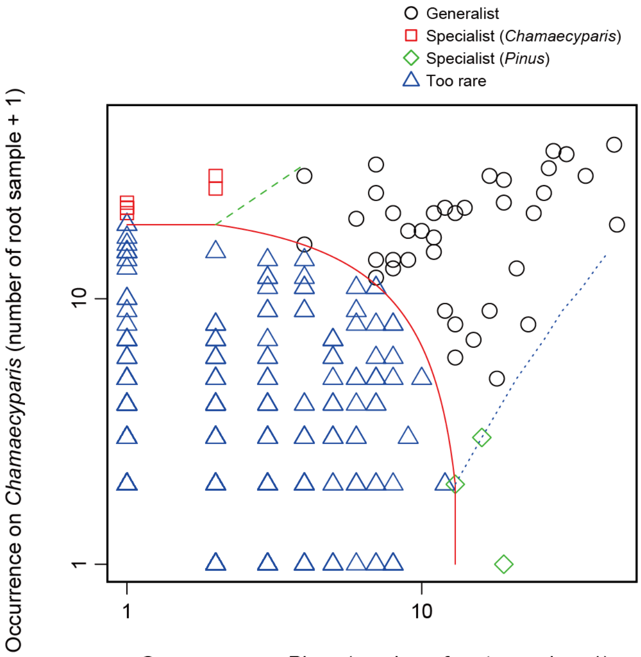

Supplementary Figure 3. Screening of specialists and generalists (analysis with equal sample size). Fungal OTUs commonly detected from both *Chamaecyparis* and *Pinus* root samples (circle), those preferentially found from *Chamaecyparis* (square) or *Pinus* (diamond) samples, and rare fungal OTUs (triangle) were classified by a CLAM test.
