# Supplementary material for: Root-Associated Fungi Shared Between Arbuscular Mycorrhizal and Ectomycorrhizal Conifers in a Temperate Forest
Source: Front Microbiol. 2018 Mar 12;9:433. doi: 10.3389/fmicb.2018.00433 (PMC5858530; doi:10.3389/fmicb.2018.00433)
Supplement: Supplementary file 9 [file Image4.PDF]

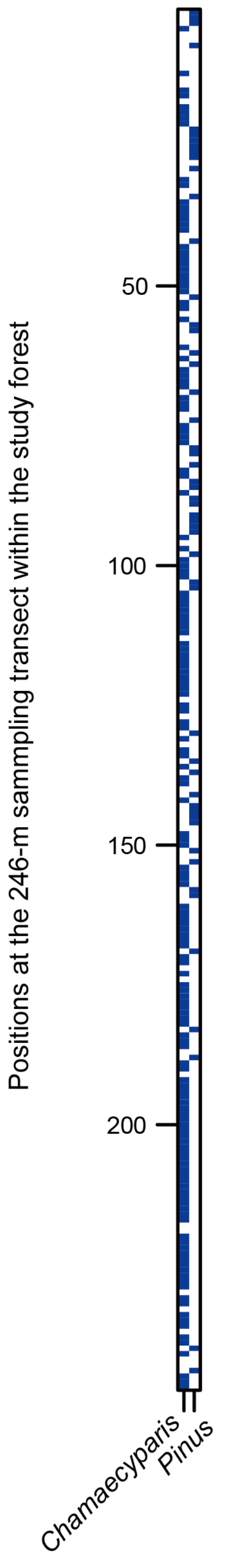

Supplementary Figure 4. Spatial autocorrelation in the occurrences of *Chamaecyparis* and *Pinus* root samples. The sampling positions along the 246-m transect in the study forest is indicated for the

Chamaecyparis and *Pinus* root samples for which sequencing reads were successfully obtained (blue squares). Outside the 246-m transect, the slant of the mountain trail was too steep to collect root samples.
